# Repertoire of P-glycoprotein drug transporters in the zoonotic nematode *Toxocara canis*

**DOI:** 10.1038/s41598-023-31556-1

**Published:** 2023-03-27

**Authors:** Jeba R. J. Jesudoss Chelladurai, Katy A. Martin, Pam Vardaxis, Craig Reinemeyer, Paramasivan Vijayapalani, Alan P. Robertson, Matthew T. Brewer

**Affiliations:** 1 Department of Veterinary Pathology, College of Veterinary Medicine, Iowa State University, Ames, IA USA; 2 Department of Diagnostic Medicine and Pathobiology, College of Veterinary Medicine, Kansas State University, 1800 Denison Ave, Manhattan, KS USA; 3 East Tennessee Clinical Research, Rockwood, TN USA; 4 Department of Biomedical Sciences, College of Veterinary Medicine, Iowa State University, Ames, IA USA; 5 Department of Veterinary Pathology, College of Veterinary Medicine, Iowa State University, 1800 Christensen, Ames, IA USA

**Keywords:** Pharmacology, Target identification, Parasite biology, Parasite physiology

## Abstract

*Toxocara canis* has a complex lifecycle including larval stages in the somatic tissue of dogs that tolerate macrocyclic lactones. In this study, we investigated *T. canis* permeability glycoproteins (P-gps, ABCB1) with a putative role in drug tolerance. Motility experiments demonstrated that while ivermectin failed to abrogate larval movement, the combination of ivermectin and the P-gp inhibitor verapamil induced larval paralysis. Whole organism assays revealed functional P-gp activity in larvae which were capable of effluxing the P-gp substrate Hoechst 33342 (H33342). Further investigation of H33342 efflux demonstrated a unique rank order of potency for known mammalian P-gp inhibitors, suggesting that one or more of the *T. canis* transporters has nematode-specific pharmacological properties. Analysis of the *T. canis* draft genome resulted in the identification of 13 annotated P-gp genes, enabling revision of predicted gene names and identification of putative paralogs. Quantitative PCR was used to measure P-gp mRNA expression in adult worms, hatched larvae, and somatic larvae. At least 10 of the predicted genes were expressed in adults and hatched larvae, and at least 8 were expressed in somatic larvae. However, treatment of larvae with macrocyclic lactones failed to significantly increase P-gp expression as measured by qPCR. Further studies are needed to understand the role of individual P-gps with possible contributions to macrocyclic lactone tolerance in *T. canis.*

## Introduction

*Toxocara canis* is a cosmopolitan zoonotic nematode parasite of dogs. *T. canis* larvae are transplacentally transferred from bitches to neonatal puppies, and after a complex hepato-pulmonary-tracheal migration, develop to adult worms in the small intestine. In older puppies and adult dogs, larvae that hatch following the ingestion of infective eggs migrate to the skeletal muscles, kidneys, liver and heart and persist for years as somatic larvae^1^. Somatic larvae in the tissues of bitches are reactivated during pregnancy and are a reservoir of infection for up to three litters following a single infection^2^. Prenatal transmission of reactivated larvae has been prevented using a few experimental drug regimens^3–5^. However, non-reactivated somatic larvae fail to be killed by macrocyclic lactones or any other anthelmintic therapy in dogs^6^. In addition, somatic larvae in paratenic hosts such as mice are not killed by ivermectin^7,8^. Larval migration to the somatic musculature or the brain in mice allowed larval survivability and protection against drugs^9^. Taken together, these studies demonstrate macrocyclic lactone tolerance by somatic *T. canis* larvae, but the mechanism of this tolerance is not well understood.

Ivermectin is a macrocyclic lactone with multiple molecular targets in nematodes^10^. Ivermectin acts on glutamate-gated chloride channels resulting in muscle paralysis, excretory-secretory (ES) pore-associated muscle paralysis, and inhibition of larval motility^11–13^. Ivermectin also potentiates the adherence of mononuclear cells and activated neutrophils to nematode cuticle^14^. However, ivermectin and other macrocyclic lactones are known substrates of Permeability glycoproteins (P-gps) that efflux xenobiotics from cells^15,16^. P-glycoproteins are encoded by genes from the ATP-binding cassette (ABC) class B1 family (ABCB1). Typically, one or two isoforms of the ABCB1 gene are expressed by the same gene locusby vertebratesIn contrast, nematodes commonly express a repertoire of ABCB1 genes and isoforms^17^. P-gp gene identification, assessment of expression, and functional studies have been carried out for some parasitic nematode species such as *Haemonchus contortus* and *Dirofilaria immitis*^18,19^. Several in vitro studies have hypothesized that P-gp substrates and inhibitors compete for efflux at one of three drug binding sites—H site, R site, or allosteric modulator site^20,21^.

We hypothesized that P-glycoproteins may play a role in larval ML tolerance in *T. canis* somatic larvae. Our rationale was that somatic larvae of *T. canis* are present at sites where macrocyclic lactones are bioavailable yet they are tolerant of treatment. In this study we investigated the P-gp gene family of *T. canis*, expression of individual P-gp genes in larval and adult parasites, and functional P-gp activity using whole organism assays.

## Methods

### Ethics statement

All experiments were conducted in accordance with the recommendations of the NIH Guide for the care and use of laboratory animals. The studies were conducted in accordance with ARRIVE guidelines and were approved by the Iowa State University Institutional Animal Care and Use Committee protocol #18,101.

### Parasites

*T. canis* adults were obtained opportunistically from naturally infected dogs. Adult worms voided in feces were washed in tap water and eggs were isolated from the uteri of female adult worms by careful dissection. Eggs were washed and incubated in 1 × phosphate buffered saline at room temperature for at least 2 weeks to allow development to the third larval (L3) stage. L3 larvae were isolated by a chemical hatching protocol modified from^22^.

### In vitro motility assays

Pools of hatched *T. canis* L3 larvae were individually transferred to 24 well plates containing RPMI1640 without antibiotics. Dilutions of ivermectin and/or verapamil (in 0.1% DMSO) were added to the wells with a final volume adjusted to 400 µL using RPMI1640. Plates were incubated at 37 °C in a cell culture incubator with 5% CO_2_ for 1 h. No drug controls consisted of vehicle (DMSO) only. Experiments were duplicated using a minimum of three larvae for each drug dilution. All larvae were tracked for 2 min and then individual larvae were selected for observation if they had hatched completely from the egg and were clearly motile. Videos of larval motility were recorded using the WormLab software (MBF Bioscience, Williston, VT) using the default settings to measure larval wavelength and area occupied by the moving larvae.

### In vitro H33342 efflux assay

The larval efflux assay was modified from previous studies^23–27^. Hatched larvae were washed in 1 × Dulbecco’s PBS and exposed to 10 µM ivermectin or P-gp inhibitors (cyclosporine A, loperamide, reserpine, verapamil or tariquidar) for 1 h at 37 °C with horizontal shaking at 200 rpm. Larvae were then incubated in 15 µM Hoechst 33,342 (H33342), a fluorescent P-gp substrate, for 10 min at 37 °C. The larvae were washed twice with 1 × D-PBS, placed on glass slides and bright-field and fluorescent images were captured using an Olympus BX 60 microscope. Images obtained by fluorescence microscopy were analyzed using the fluorescence area module in Halo (Indica Labs, Advanced Cell Diagnostics, Hayward, CA). Outline of each larva for this measurement was obtained from the bright field images. Percentage of the area stained by H33342 in larvae was calculated using the formula:$\left( {{\text{Fluorescently}}\;{\text{ stained }}\;{\text{area}}/{\text{ Total}}\;{\text{ area}}\;{\text{ of}}\;{\text{ larva}}} \right) \times {1}00.$

### Genomic survey and phylogenetic analysis

The 317 Mb draft genome of *T. canis*^28^ on NCBI (*Toxocara canis* isolate PN_DK_2014, whole genome shotgun sequencing project, GenBank Accession JPKZ00000000 was surveyed, and we identified and annotated putative P-gp genes. All were previously annotated as either pgp-1 or pgp-3. Nucleotide sequences of P-gp genes of *T. canis* retrieved from GenBank were used to design custom primers to amplify partial Pgp sequences from adult cDNA. PCR reactions consisted of 1 × OneTaq HotStart DNA polymerase master mix (New England BioLabs), 1 µM of each primer and 2 µL of cDNA and amplified using touch-down PCR. Amplicons were visualized on an agarose gel, purified, and cloned into pCR-XL-TOPO for sequencing on an Applied Biosystems 3730xl DNA analyzer at the Iowa State University DNA Facility. Seven amplified nucleotide sequences were conceptually translated to protein sequences for phylogenetic analyses. Protein sequences translated from nucleotide sequences of P-gp genes described in other nematodes such as *Haemonchus contortus*, *Cooperia oncophora*, *Teladorsagia circumcinta*, *Cylicocyclus* spp., *Parascaris* spp*., Dirofilaria immitis*, and *C. elegans* were obtained from GenBank and aligned with the MAFFT algorithm^29^. Substitution model was selected using the SMS tool with Bayesian Information Criteria^30^. The best ML model was LG + F + I + G with 4 parameter gamma distribution^31^. Maximum likelihood phylogenetic analyses were performed using PhyML3.0^32^. The tree was visualized using Mega X^33^.

### qPCR in adult *Toxocara canis*

Expression levels of P-gp genes was determined using qPCR in adult male and female worms. qPCR primers were designed to amplify the genes (Table S1) and specificity was confirmed using BLAST in silico. RNA extracted from pools of adult worms and cell types was extracted with Trizol reagent followed by purification using the Direct-zol RNA Miniprep kit (Zymo Research) according to the manufacturer's instructions. cDNA was synthesized from 50 ng of total RNA in a 20 µL volume using the iScript cDNA synthesis kit (Bio-rad), using random oligonucleotides. qPCR reactions were individually optimized using diluted cDNA synthesized from adult *T. canis* worms. Specificity was determined using melt curve analysis and sequencing. 18 s RNA was used as a reference gene and amplified using previously described primers^34^. qPCR was carried out in technical duplicates in a volume of 20 µL with 2 µL of diluted cDNA, 1 × of SSoAdvanced Universal SYBR Green Master Mix (Bio-rad) and 0.2–0.5 µM of diluted primers. PCR efficiency was determined for each primer pair using LinRegPCR^35^ and change in gene expression was calculated using the efficiency corrected ΔCt method based on single samples^36^.

### qPCR following in larvae exposed to drugs in vitro

Following hatching, pools of 500 L3 were washed in 1 × Dulbecco’s PBS and exposed to 10 µM Ivermectin, Moxidectin (MP Biomedicals, Solon, OH) or no drugs (control) in RPMI 1640 (Gibco) at 37 °C with horizontal shaking at 200 rpm for 24 h. Drug exposure assays were carried out with three different isolates of *T. canis* eggs. Immediately after drug exposure, larvae were washed in 1X DPBS and transferred to sterile RPMI1640. Sterile 0.1 mm, 0.5 mm and 2 mm Zymo bashing beads (Zymo research, Irvine, CA) were used to homogenize larvae in Trizol reagent and total RNA was extracted following the manufacturer’s protocol. RNA concentration and purity were measured using a Nanodrop spectrophotometer and stored at −80 °C. cDNA was synthesized from 50 ng of total RNA in a 20 µL volume using the iScript cDNA synthesis kit (Bio-rad) using random oligonucleotides and stored at −20 °C till use. To determine differences between drug exposed and unexposed larvae, qPCR was carried out in technical duplicates in a volume of 20 µL with 2 µL of undiluted cDNA, 1 × of SSoAdvanced Universal SYBR Green Master Mix and 0.2–0.5 µM of diluted primers. PCR efficiency was determined for each primer pair using LinRegPCR^35^. Fold changes in gene expression were calculated using the efficiency corrected ΔCt method based on single samples^36^.

### qPCR following in vivo drug exposure in mice

Expression of P-gp genes was measured in *T. canis* larvae recovered from treated and untreated mice. Experiments were conducted in C3H/HEJ mice (Jackson Labs) with 3 males and 3 females per treatment group, and the study was conducted twice using different batches of mice. Mice were gavaged with 5000 larvated *T. canis* eggs and housed in a ventilated rack cage system with standard enrichment. Mice were injected subcutaneously with ivermectin (200 µg/kg), moxidectin (500 µg/kg), or untreated on day 7 post-infection and euthanized on day 10 post-infection. Liver, lungs, and brain were collected from each mouse and frozen at − 80 °C for RNA extraction. Somatic larvae were obtained by pepsin digestion of tissue followed by isolation of larvae using a membrane filter (Millipore). Total RNA was then extracted using Trizol. qPCR following cDNA synthesis was performed using the methods outlined above for *T. canis* adult and larvae.

### RNAscope in situ hybridization

Hatched *T. canis* larvae were fixed in 10% neutral buffered formalin for at least 24 h and embedded in warm histogel (Fisher Scientific) by pipetting the larval pellet into a 1 cm^3^ plastic mold containing liquid histogel and allowing the cube to dry. Solidified histogel blocks were embedded in paraffin. Paraffin sections of 5 µm thickness were mounted on SuperFrost slides (Fisher Scientific) and dried for 1 h at 60 °C. In situ hybridization probes targeting *T. canis P-gp 11* (JPKZ01003065), *P-gp 2* (JPKZ01001761) and β*-tubulin* (JPKZ01000754.1) were designed and provided by Advanced Cell Diagnostics (Hayward, CA). A probe targeting *T. canis β-tubulin* was used as a positive control. A probe targeting *dapB* of *Bacillus subtilis* was used as a negative control. RNAscope 2.5 HD Duplex kit (Green/Red, Advanced Cell Diagnostics) was used to detect mRNA transcripts and target sequences of the probes are listed in Table S2. Slides processed according to the manufacturer’s instructions. Hybridization amplification steps were carried out to detect green signal. Slides were counterstained with 50% Hematoxylin and mounted with Vectashield antifade mounting medium (Vector Laboratories). Expression of mRNA was analyzed in an Olympus BX53 microscope and images were captured with an Olympus DP73 camera using Olympus CellSens Dimension software. Images were processed using Adobe Photoshop (*version* 2020).

### Statistical analysis

One-way ANOVA with Tukey’s multiple comparison test was used to compare gene expression and motility between different experimental groups using GraphPad Prism version 9 (San Diego, CA).

## Results

### Ivermectin-induced paralysis of larvae observed following P-gp inhibition

**Figure 1:**
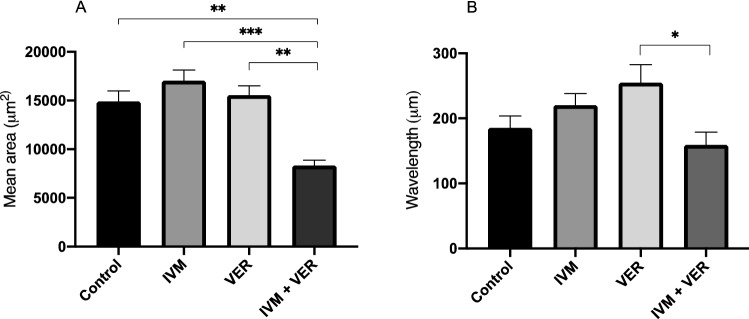
Ivermectin-induced paralysis of larvae is only observed following P-gp inhibition. Bars represent (**A**) mean area occupied or (**B**) mean wavelength ± SE of larvae following exposure to ivermectin with or without verapamil. (Asterisks indicate groups that are significantly different, ***p < 0.001, **p < 0.01, *p < 0.05).

*T. canis* larvae exhibited sinusoidal thrashing motility in liquid media without progressive or forward movement. The WormLab software tracked moving larvae using anterior, middle and posterior markers. The mean area occupied by larvae was significantly reduced after incubation with a combination of verapamil and ivermectin, but not ivermectin alone (Fig. 1A). A decrease in area occupied during the drug trial corresponds to a decrease in overall larval motility. The Wormlab software also measured the wavelength of sinusoidal larval movement so we hypothesized that the wavelength would decrease as ivermectin caused paralysis. Larvae treated with the drug combination also had decreased wavelength but not ivermectin alone (Fig. 1B). Thus, in the WormLab system, P-gp inhibition appeared to be required to induce paralysis in the presence of macrocyclic lactones such as ivermectin.

### Unique pharmacological profile of P-gp inhibition in *T. canis* larvae

**Figure 2:**
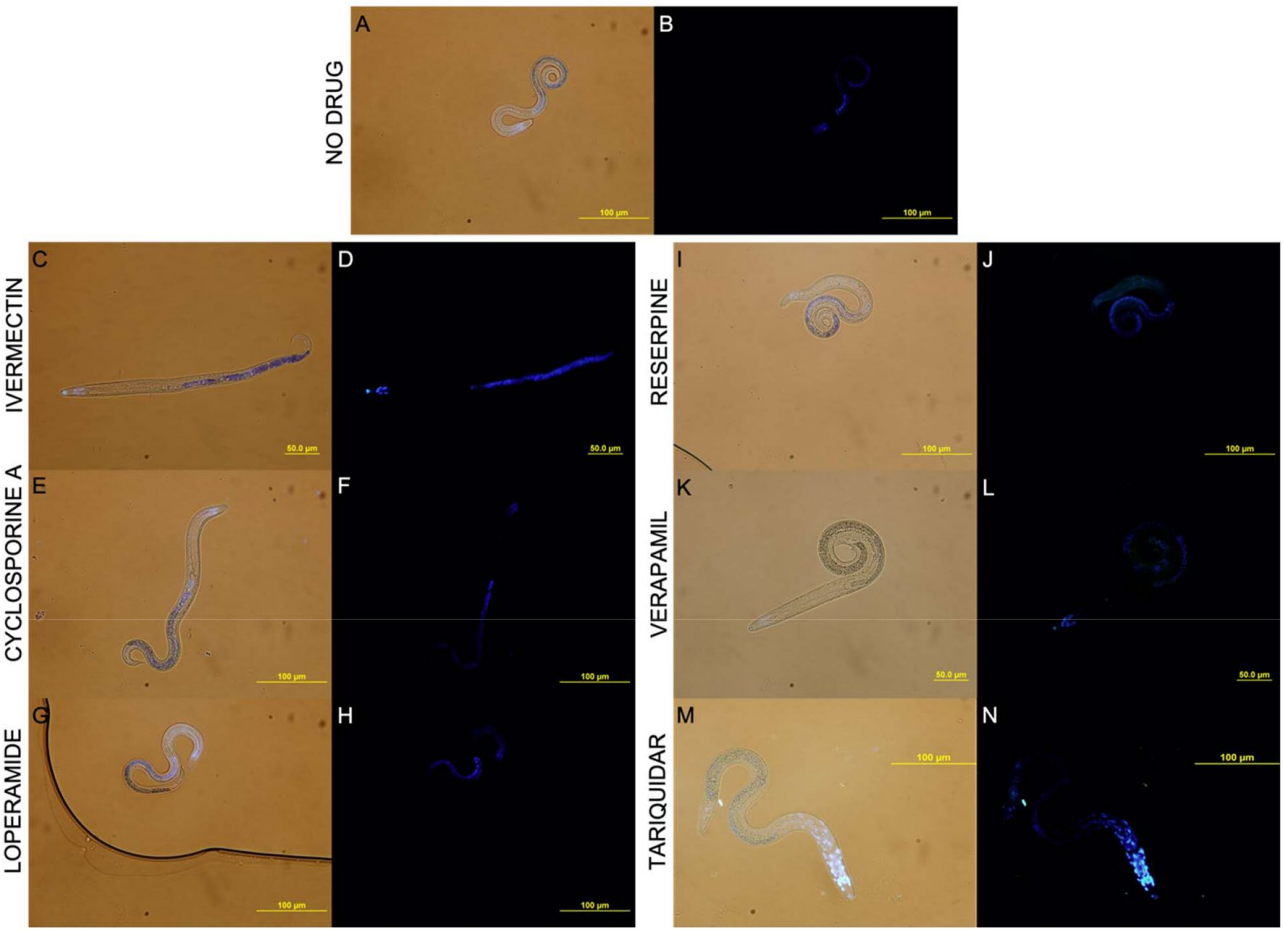
Representative bright-field overlays and fluorescence images of T. canis larvae stained with Hoechst 33,342 in the absence of drugs (**A**, **B**) and presence of inhibitors-ivermectin (**C**,**D**), cyclosporine A (**E**, **F**), loperamide (**G**, **H**), reserpine (**I**, **J**), verapamil (**K**, **L**) and tariquidar (**M**, **N**) (400x). The brightfield image was used to annotate the outline of the larvae which was overlaid on the fluorescent image. The area stained was quantitated using image analysis software (Fig. 3).

**Figure 3:**
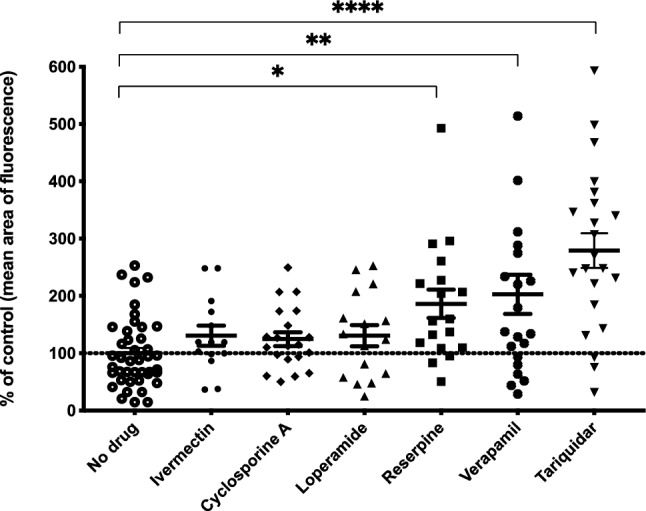
Increased H33342 staining of T. canis larvae exposed to P-gp inhibitors. Mean ± SE are shown, asterisks indicate P-gp inhibition relative to no drug control. (Asterisks indicate groups that are significantly different, ****p < 0.0001, **p < 0.01, *p < 0.05).

Total larval P-gp activity and inhibition was measured by a Hoechst 33,342 efflux assay^27^. H33342 fluoresces within cells but not after P-gp has effluxed this substrate out of the plasma membrane^37^, allowing fluorescence microscopy of larvae without background signal on a slide. Larvae were treated with and without P-gp inhibitors, photographed (Fig. 2), and positive H33342 staining was quantitated using Halo (Advanced Cell Diagnostics, Hayward, CA). computer software (Fig. 3). Untreated larvae had constitutive P-gp efflux activity, indicated by the absence of H33342 staining (Fig. 2A,B). In contrast, larvae treated with P-gp inhibitors had reduced efflux activity leading to retention of H33342 (Fig. 2). Notably, P-gp-mediated efflux was sensitive to reserpine, verapamil, and tariquidar, but not ivermectin, cyclosporine A, or loperamide (Fig. 3).

### Genomic analysis reveals at least thirteen P-gp genes identified in *T. canis*

**Figure 4:**
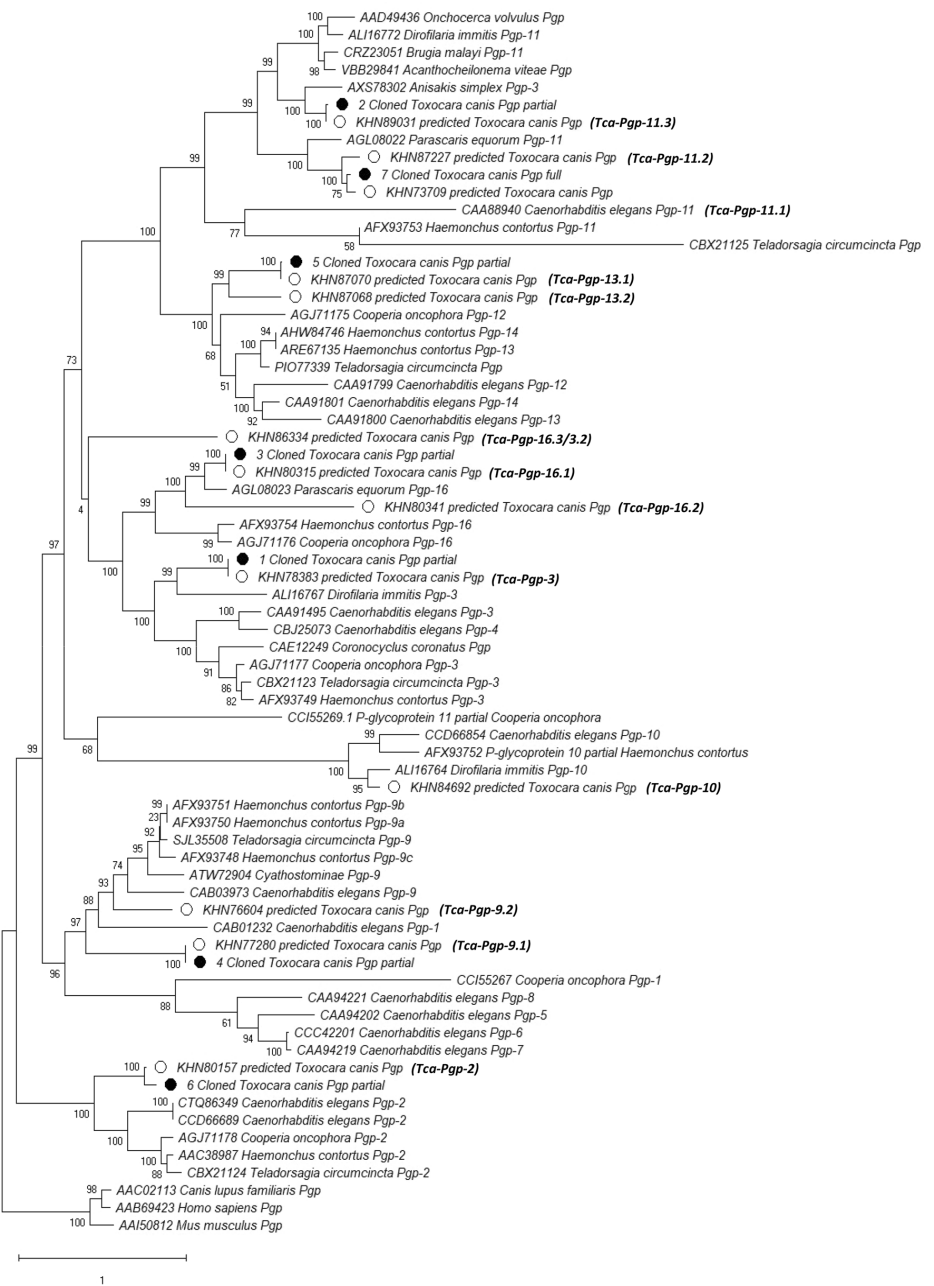
Maximum likelihood phylogenetic tree of conceptually translated P-gp genes. Pgp genes predicted in the genome (o) and cloned ($\bullet$) are highlighted along with assigned names for T. canis P-gp genes.

The draft genome of *Toxocara canis* assembly GCA_000803305.1 on the NCBI Genome database was used for bioinformatic analysis and primer design. Thirteen P-gp protein sequences with length > 200 amino acids (at least 1/6th the expected length of a Pgp) annotated in the *T. canis* genome were identified. Gene sequences were also obtained from the cloning of six partial (> 1000 nucleotides) and one full length Pgp (3849 nucleotides) from mRNA. Conceptually translated amino acid sequences from P-gp genes annotated in the *T. canis* genome, annotated in genomes of other nematodes obtained from GenBank, and cloned sequences obtained in this study were used in the Maximum Likelihood phylogenetic analyses (Fig. 4). Sequences were deposited in GenBank (MT495501-MT495506, MT543030). This enabled assigning gene names to the 13 P-gps (Table 1). Based on the phylogenetic analysis, several of these were determined to be paralogs. Isoforms have been observed in other nematode P-gps including *Haemonchus contortus* Pgp-9^38^. However, since the genes identified in Fig. 4 are annotated in the nuclear genomes, we have designated them as paralogs rather than isoforms which are transcribed from the same genetic locus. Our analysis revealed three annotated paralogs of Pgp-11 and two paralogs for Pgp-9, Pgp-13 and Pgp-16. One P-gp (KHN86334) has an ambiguous position with low statistical support in the tree and was named (*Tca-Pgp-16.3/3.2*).

**Table 1:** Nomenclature for P-gp protein sequences in Toxocara canis.

GenBank accession number (Protein database)	Length of annotated protein	Assigned gene names
KHN80157	1285aa	Tca-Pgp-2
KHN86334	1700aa	Tca-Pgp-16.3/3.2
KHN78383	1352aa	Tca-Pgp-3
KHN77280	741aa	Tca-Pgp-9.1
KHN76604	462aa	Tca-Pgp-9.2
KHN84692	1368aa	Tca-Pgp-10
KHN73709	1337aa	Tca-Pgp-11.1
KHN87227	1337aa	Tca-Pgp-11.2
KHN89031	1268aa	Tca-Pgp-11.3
KHN87070	1355aa	Tca-Pgp-13.1
KHN87068	1330 aa	Tca-Pgp-13.2
KHN80315	1368aa	Tca-Pgp-16.1
KHN80341	2130aa	Tca-Pgp-16.2

### Life cycle stage-specific expression of P-gps

**Figure 5:**
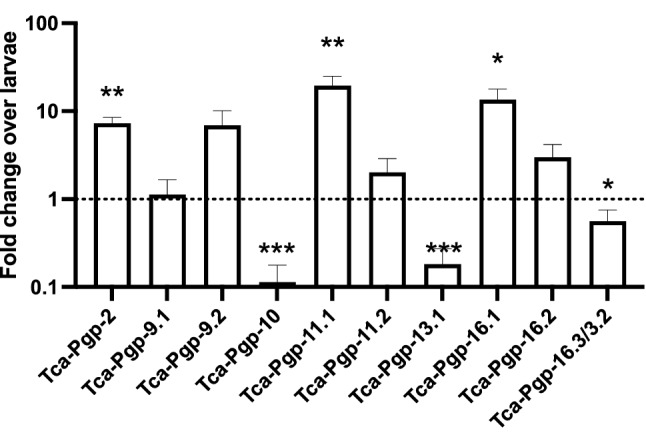
Fold change ± SE in expression of Pgp genes in adult T. canis worms compared to hatched larvae. Fold change was calculated using the efficiency corrected ΔCt method using T. canis 18S as the reference gene. Bars above the dotted line represent increased transcription in adults and bars below the line represent increased transcription in larvae. (Asterisks represent statistically significant differences, ***p < 0.001, **p < 0.01, *p < 0.05).

Constitutive expression levels of P-gp genes were determined using qPCR in adult *T. canis* nematodes and infective larvae hatched from eggs. P-gp expression was detected for 10 genes or paralogs of the predicted genes (Fig. 5). Specific primer sets could not be designed/optimized for *Tca-Pgp-3*, *Tca-Pgp-11.3* and *Tca-Pgp-13.2* because of very high sequence cross-identity and the presence of multiple peaks in melt curve analyses of products, reinforcing our finding that paralogs of the genes exist as predicted in the phylogenetic analysis. *Tca-Pgp-2*, *Tca-Pgp-11.1* and *Tca-Pgp-16.1* were significantly upregulated in the adults compared to infective larvae (*q* < 0.01, Fig. 5). On the other hand, *Tca-Pgp-10* and *Tca-Pgp-13.1* had decreased transcription levels compared to larvae.

### Changes in P-gp expression following exposure of larvae to macrocyclic lactones

**Figure 6:**
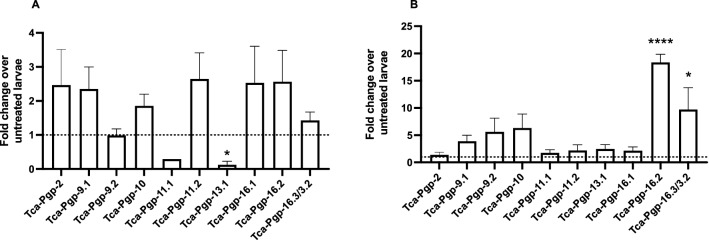
Mean fold change ± SE in expression of P-gp genes following (**A**) ivermectin or (**B**) milbemycin oxime treatment of larvae hatched in vitro. Fold change was obtained using the efficiency corrected ΔCt method using T. canis 18S as the reference gene. (Asterisk represents statistical difference from untreated controls ****p <  < 0.0001, *p < 0.05).

Expression profile changes for 10 P-gp genes and paralogs in *T. canis* larvae were determined after treatment with 10 µM ivermectin or milbemycin oxime for 24 h (Fig. 6A). *Tca-Pgp-16.2* and *Tca-Pgp-16.3/3.2* were significantly upregulated in larvae treated with milbemycin oxime compared to untreated larvae (*q* < 0.01, Fig. 6B). *Tca-13.1* was downregulated in the presence of ivermectin (*p* < 0.05, Fig. 6A). Other statistically significant expression changes were not detected during the time points used in this study.

### Effect of macrocyclic lactone treatment on larval P-gp expression in vivo

**Figure 7:**
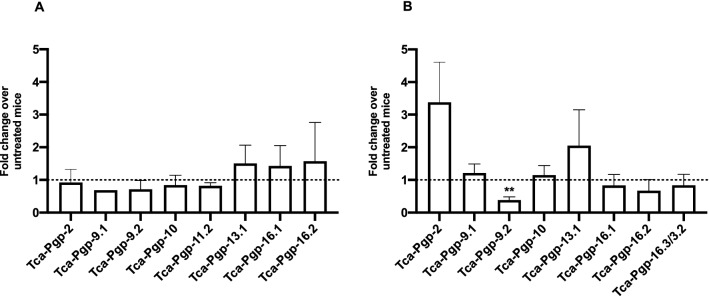
Mean fold change ± SE in expression of P-gp genes in larvae from mice treated with (**A**) ivermectin or (**B**) moxidectin compared to untreated controls. Fold change was calculated using the efficiency corrected ΔCt method using T. canis 18S as the reference gene. (Asterisk represents statistical difference from untreated controls **p < 0.01).

Expression profile of P-gp genes was determined in somatic *T. canis* larvae derived from infected mice. P-gp expression was detected in somatic larvae for 8 genes or paralogs of the predicted genes (Fig. 7). There were no statistical differences among genes in the ivermectin-treated group, while *Tca-Pgp-9.2* was significantly downregulated in larvae from moxidectin-treated mice (*p* < 0.05, Fig. 7B). *Tca-Pgp-2* appeared to be upregulated in larvae from moxidectin-treated mice, but this was not statistically significant (*p* = 0.0693). Several P-gp genes and paralogs could not be amplified from somatic larvae, possibly due to overabundance of host RNA or low levels of gene expression. Innovative techniques that can retrieve high numbers of *T. canis* larvae from the host will help progress these types of studies in the future.

### Detection of *P-gp* expression in larval tissues

Expression of *P-gp* mRNA in the in vitro hatched *T. canis* larvae was analyzed by RNAscope assays. For this experiment, we focused on Tc-Pgp-11, which is a sequelogue of other significant nematode P-gps that has been more extensively characterized (Jesudoss 2021).

**Figure 8:**
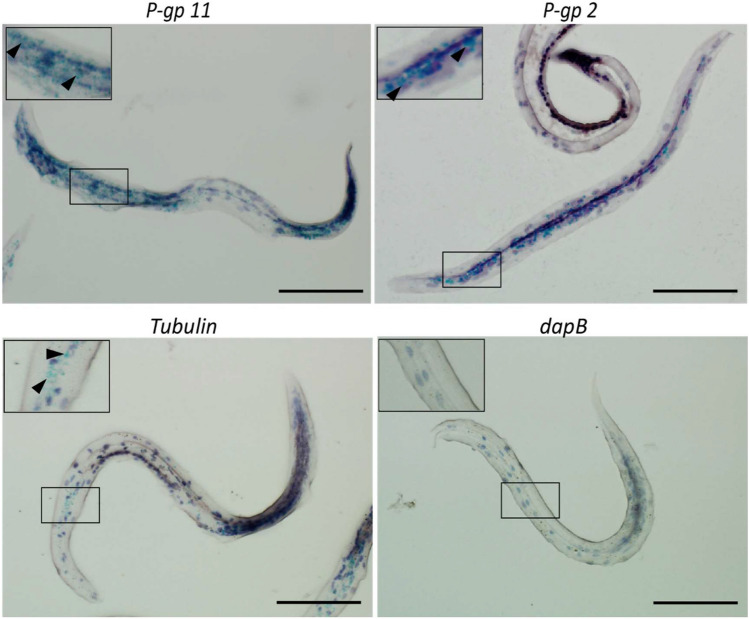
Expression of P-gp mRNA in T. canis larvae in situ detected by RNAscope assay. Small box indicates location of magnified inset. Hybridization of probe (green punctates) is indicated by arrow heads. T. canis β-tubulin (positive control) and Bacillus subtilis dapB (negative control) were probed in parallel. Scale bar = 50 μm.

Abundant localization of green puncta corresponded to P-gp expression in the nematode intestine whereas *β-tubulin* was detected throughout the larvae (Fig. 8). The negative control *dapB* probe did not result in any hybridization signal (Fig. 8). The detection of P-gp transcripts in the intestine is consistent with our other observations in ascarids (Jesudoss 2019, Jesudoss 2021). Further investigation is required to confirm that other *T.* canis P-gps are expressed in the intestinal cells.

## Discussion

Bitches harboring somatic *T. canis* larvae are reservoirs for infection to puppies, which occurs primarily by a transplacental route. Arrested somatic larvae evade drug-mediated killing until they are reactivated during the third trimester of pregnancy, but the reason for this drug-tolerant phenotype in non-reactivated somatic larvae has not been elucidated. P-glycoprotein efflux pumps are one of several possible mechanisms contributing to tolerance. In this study, we investigated the gene family, mRNA transcription, and functional relevance of P-gps in *T. canis*.

Motility has been used to determine the activity of anthelmintics against different stages of nematodes^39–41^. Clade V nematodes such as the trichostrongyles show motility responses when ivermectin is added to larvae in vitro^42^. Interestingly, we observed that ivermectin treatment did not alter motility of *T. canis* larvae, a clade III nematode. This was not unexpected as many studies have shown that in clade III nematodes exposure to physiological levels of macrocyclic lactones fails to inhibit larval motility, thus supporting the hypothesis that ivermectin exerts an immune-mediated effect in addition to paralysis^14^. In the present study, global inhibition of nematode P-gp using verapamil was required for ivermectin-induced paralysis of larvae. Our findings are in agreement with other studies demonstrating the potentiation of ivermectin with P-gp inhibiting drugs^43–45^. Due to the type of thrashing motility exhibited by *T. canis* larvae, we moved on in favor of fluorescent efflux assays for further assessment of P-gp activity in larvae.

An important finding of this study was that *T. canis* larvae efflux the P-gp substrate H33342, indicating the presence of constitutive P-gp activity. This activity could be abrogated by the P-gp inhibitors tariquidar, verapamil, and reserpine. Interestingly, numerous other known inhibitors of mammalian P-gp failed to inhibit H33342 efflux in *T. canis* larvae. In particular, it was surprising that ivermectin did not appear to inhibit P-gp efflux in larvae using the H33342 assay. We hypothesize that the kinetics of H33342 efflux vary for different substrates and the timing for our photomicrographic assays was not sensitive to capture this. In addition, H3342 efflux may be carried out by other members of the ATP-binding cassette transporter family that are insensitive to ivermectin.

Although our assays represent global P-gp activity, these experiments suggest that *T. canis* P-gps exhibit a unique pharmacology that is likely to be nematode-specific. Although the H33342 assay provides a useful demonstration of P-gp mediated efflux, individual P-gp genes need to be studied in order to discover the unique pharmacological profile for each protein.

Analysis of the *T. canis* draft genome led to an estimate of 530 genes that were likely to be transporters (out of the 18,596 protein coding genes), of which 10.8% were predicted to be ABC transporters by computer algorithms ^28^. Of all the sequences annotated as P-gp genes in the genome of *T. canis*, our phylogenetic analysis suggests that only 13 genes likely exist, of which several are paralogs. > 1000 base pair fragments of seven of these genes could be cloned from mRNA, suggesting that they are transcribed genes. Gene naming conventions based on *C. elegans* genes as suggested by^46^ were used except where cases of ambiguity existed such as *Tca-Pgp-16.3/3.2.* Uncertainties in the assigning of genes can be resolved as genome assemblies of organisms improve and as paralogs are studied in closely related nematodes.

Our study reveals a repertoire of P-gp genes expressed by both larval and adult stages of the parasite that merit further pharmacological characterization. Our results also suggest that larvae hatched in vitro are useful as a surrogate for, but not replacement of, studies of somatic larval *T. canis.* We detected mRNA transcripts from at least 10 of the 13 named genesexpressed in *T. canis* adults and larvae in this study. While comparisons of adults and hatched infective larvae showed that several genes were upregulated or downregulated, the importance of constitutive expression of even low levels of P-gps may have functional significance in both life stages. In larvae exposed to ivermectin in vitro for 24 h, downregulation of *Tca-Pgp-13.1* occurred, but no other changes were seen in other genes. In larvae exposed to milbemycin oxime, *Tca-Pgp-16.2* and *Tca-Pgp-16.3/3.2* were significantly upregulated. It is possible that larval gene regulatory changes occur earlier or later than 24 h and were not detected at the 24 h time point. It is also possible that the high doses of ivermectin affected the worms such that transcription could no longer occur. This is a limitation of this study that needs to be clarified in further nematode P-gp research projects. It will be informative to map fine scale temporal gene expression changes that result from drug exposure with assays that provides greater molecular resolution such as RNA-seq. Such studies necessitate a range of variables such as drug dose and incubation time.

P-gp gene mRNA expression was also detected in *T. canis* somatic larvae derived from the liver, brain and lungs of experimentally infected mice. Mice are natural paratenic hosts for *T. canis* and are a tractable model for studying somatic larval migrans of humans. Ivermectin at various doses and routes is unable to eliminate larvae in mice^7^. Although our study did not detect any specific P-gp gene that was significantly upregulated following treatment, our study demonstrates that at least 8 P-gp genes or isoforms are expressed by larvae in the somatic tissues. It is possible that functional drug efflux by these P-gps contributes to macrocyclic lactone tolerance in *T. canis* larvae. Changes in P-gp expression were not detected following treatment of mice, however, this could be due to interference by host RNA or the amount of parasite RNA retrieved from individual granulomas. Methods for recovery of *T. canis* larvae from somatic tissue of hosts is needed to further investigate larval gene expression in vivo.

This study yielded significant findings regarding P-gps from the zoonotic nematode *T. canis*. We curated and named 13 P-gp genes present in the genome which was supported by phylogenetic analysis and molecular cloning. Experiments demonstrated phenotypic evidence that P-gp inhibition potentiates ivermectin-induced larval paralysis, and that *T. canis* larvae exhibit functional P-gp efflux activity. Importantly, functional inhibition studies using larvae suggest that *T. canis* P-gps have a unique pharmacological profile. This highlights the potential of nematode P-gps as parasite-specific drug targets, however, the inhibition profile needs to be investigated for each individual nematode P-gp in isolation. Another significant finding was confirmation of constitutive P-gp expression for numerous P-gp genes in adult worms, larvae hatched in vitro, and somatic larvae recovered from mice. In conclusion, *T. canis* expresses a large repertoire of P-gps which are functional efflux proteins and appear unresponsive to several traditional inhibitors of mammalian P-gp. Taken together, our findings support further study of the P-gp family and other transmembrane transporters in the search for nematode-specific drug targets.

## Supplementary Information

Supplementary Information.

## Data Availability

The datasets generated during the current study are available from the corresponding author upon reasonable request. Gene sequences are deposited under GenBank accession #MT495501-MT495506, MT543030.
