# Supplementary material for: Repertoire of P-glycoprotein drug transporters in the zoonotic nematode Toxocara canis
Source: Sci Rep. 2023 Mar 27;13:4971. doi: 10.1038/s41598-023-31556-1 (PMC10042841; doi:10.1038/s41598-023-31556-1)
Supplement: Supplementary file 1 — Supplementary Information. [file 41598_2023_31556_MOESM1_ESM.pdf]

Table S1. qPCR primers used in this study

Gene name	Primer sequence
Tca-Pgp- 2	5’ ATGCGATCTAGACAAGTTGAGG
	5’ TCAGGCCACTGCAACAAGTG
Tca-Pgp-9.1	5’ AGGGCGGTATGAGAGATGGT
	5’ CGAGCGCATAAGAGCCAAAC
	5’ GACGACACGCAGCATATAATTC
	5’ GATTGGTGTTGTATCGCAAGAG
Tca-Pgp-9.2	5’ GCTATAACAAGGACGCGAAGG
	5’ TGAATTTTTGTGCGGTCTTTCC
Tca-Pgp-10	5’ TGGTCATGGCTTCTGTGGTC
	5’ AGACGACCTGGAGTGTTCCT
Tca-Pgp-11.1	5’ TGCAACGGTCAGGAAACGAT
	5’ AATATCGCCCGGCTCTTTGA
Tca-Pgp-11.2	5’ TGCAAGAGGCGCTCAAAAAG
	5’ AAGCGACGACAAGCGATGAG
Tca-Pgp-13.1	5’ GAGGACGTATCATCGAACAAGG
	5’ GAGTTGAAACTGTTGGGCTTTC
Tca-Pgp-16.1	5’ GGATATGACCGAGGAAGAAATGG
	5’ GCACGTGCGATAGCGATAC
Tca-Pgp-16.2	5’ GCTCGTGTTGAAATGTGGGAG
	5’ GTTCTTGACCGTTGTAAGCGA
Tca-Pgp-16.3/3.2	5’ TGCAGATCCAGGTGAAACGA
	5’ TCCCGAGACGGCATCATAGT
Tca-18S	5’ CTACCACATCCAAGGAAGGCA
	5’ TTATTTTTCGTCACTACCTCCTCATG

Table S2. Probes used in RNAscope assay

Probe	GenBank Accession	Target nucleotide
Tca-P-gp-11.1	JPKZ01003065	955-2174
Tca-P-gp-2	JPKZ01001761	331-1276
Tca-β-tubulin	JPKZ01000754.1	65-1311
Bacillus subtilis dapB	EF191515	414-862
